# Electrochemical data of Co(II) complexes containing phenanthroline functionalized ligands

**DOI:** 10.1016/j.dib.2018.10.046

**Published:** 2018-10-19

**Authors:** Hendrik Ferreira, Marrigje M. Conradie, Jeanet Conradie

**Affiliations:** Department of Chemistry, University of the Free State, PO Box 339, Bloemfontein 9300, South Africa

## Abstract

The data presented in this paper are related to the research article entitled “*Electrochemical properties of a series of Co(II) complexes, containing substituted phenanthrolines*” (Ferreira et al., 2018) [1]. This paper presents detailed electrochemical data of eight octahedral Co(II) complexes containing functionalized phenanthrolines-ligands. The data illustrate the shift in the Co^III/II^ and Co^II/I^ redox couples due to different substituents on the phenanthrolines. Polypyridine Co(II) and Co(III) complexes exhibit properties as potential mediators in dye-sensitized solar cells (DSSCs) (Gajardo and Loeb, 2011; Yu et al., 2011) [2], [3]. The ability of a compound to act as a redox mediator to be used in DSSC, depends on the redox potential of the compound (Grätzel, 2005) [4]. Accurate data of the Co^III/II^ redox couple is presented here.

**Specifications table**

**Table:** 

Subject area	*Chemistry*
More specific subject area	*Electrochemistry*
Type of data	*Table, text file, graph, figure*
How data was acquired	*BAS 100B/W electrochemical analyzer (Electrochemical studies).*
Data format	*Raw and Analyzed.*
Experimental factors	*Samples was used as synthesized. The solvent-electrolyte solution in the electrochemical cell was degassed with Ar for 10 min, the sample was added, the sample-solvent-electrolyte solution was then degassed for another 2 min and the cell was kept under a blanket of purified argon during the electrochemical experiments.*
Experimental features	*All electrochemical experiments were done in a 2 ml electrochemical cell containing three-electrodes (a glassy carbon working electrode, a Pt auxiliary electrode and a Ag/Ag*^*+*^*reference electrode), connected to a BAS 100B/W electrochemical analyzer. Data obtained were exported to excel for analysis and diagram preparation.*
Data source location	*Department of Chemistry, University of the Free State, Nelson Mandela street, Bloemfontein, South Africa.*
Data accessibility	*Data is with article.*
Related research article	*Hendrik Ferreira, Marrigje M. Conradie and Jeanet Conradie, Electrochemical properties of a series of Co(II) complexes, containing substituted phenanthrolines, Electrochimica Acta 292 (2018) 489–501.*DOI:10.1016/j.electacta.2018.09.151.

**Value of the data**

• This data provide cyclic voltammograms and detailed electrochemical data for a comprehensive series of eight functionalized phenanthroline-Co(II) complexes, for scan rates over two orders of magnitude (0.05–5.0 V s^−1^).
• This data illustrate the influence of differently functionalized phenanthroline ligands on the redox potential of the metal they are coordinated to.
• This data illustrate that up to three reversible redox couples can be obtained in acetonitrile as solvent for tris(1,10-phenanthroline)Cobalt(II) and differently functionalized phenanthroline-Co(II) complexes.

## 1 Data

**Fig. 1:**
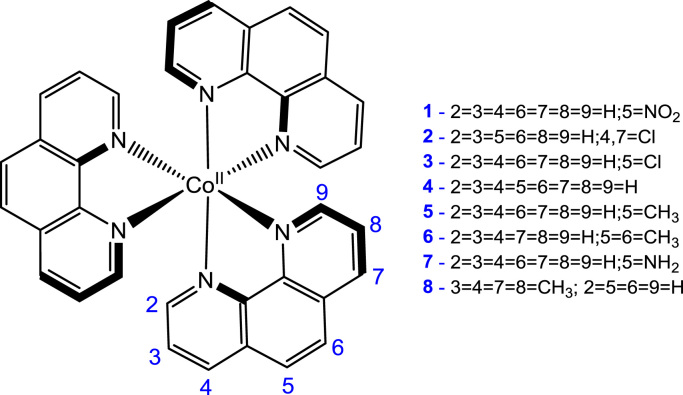
Structure and complex numbering of the functionalized phenanthroline-Co(II) complexes.

The data presented in this paper are related to the research article entitled “*Electrochemical properties of a series of Co(II) complexes, containing substituted phenanthrolines*” [1]. This paper presents detailed electrochemical data of eight octahedral Co(II) complexes containing functionalized phenanthrolines-ligands. Polypyridine Co(II) and Co(III) complexes exhibit properties as potential mediators in dye-sensitized solar cells (DSSCs) [2], [3]. The ability of a compound to act as a redox mediator to be used in DSSC, depends on the redox potential of the compound [4]. The data of the eight functionalized phenanthroline-Co(II) complexes, namely tris(5-nitro-1,10-phenanthroline)Cobalt(II) nitrate, [Co(5-NO_2_-phen)_**3**_](NO_3_)_2_ (**1**), tris(4,7-dichloro-1,10-phenanthroline)Cobalt(II) nitrate, [Co(4,7-di-Cl-phen)_**3**_](NO_3_)_2_ (**2**), tris(5-chloro-1,10-phenanthroline)Cobalt(II) nitrate, [Co(5-Cl-phen)_**3**_](NO_3_)_2_ (**3**), tris(1,10-phenanthroline)Cobalt(II) nitrate, [Co(phen)_**3**_](NO_3_)_2_ (**4**), tris(5-methyl-1,10-phenanthroline)Cobalt(II) nitrate, [Co(5-Me-phen)_**3**_](NO_3_)_2_ (**5**), tris(5,6-dimethyl-1,10-phenanthroline)Cobalt(II) nitrate, [Co(5,6-di-Me-phen)_**3**_](NO_3_)_2_ (**6**), tris(1,10-phenanthroline-5-amine)Cobalt(II) nitrate, [Co(5-NH_2_-phen)_**3**_](NO_3_)_2_ (**7**) and tris(3,4,7,8-tetramethyl-1,10-phenanthroline)Cobalt(II) nitrate, [Co(3,4,7,8-Me-phen)_**3**_](NO_3_)_2_ (**8**), is presented in this contribution, see Fig. 1 for the structures of **1**–**8**.

**Fig. 2:**
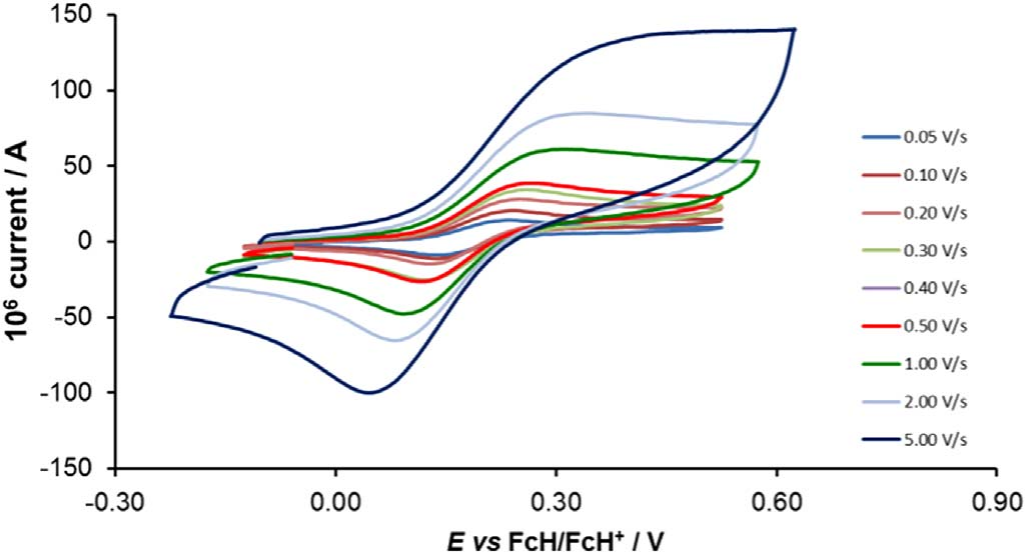
Cyclic voltammograms of complex 1 at scan rates of 0.05 V s^−1^ (lowest peak current) −5.00 V s^−1^ (highest peak current). All scans initiated in the positive direction.

**Fig. 3:**
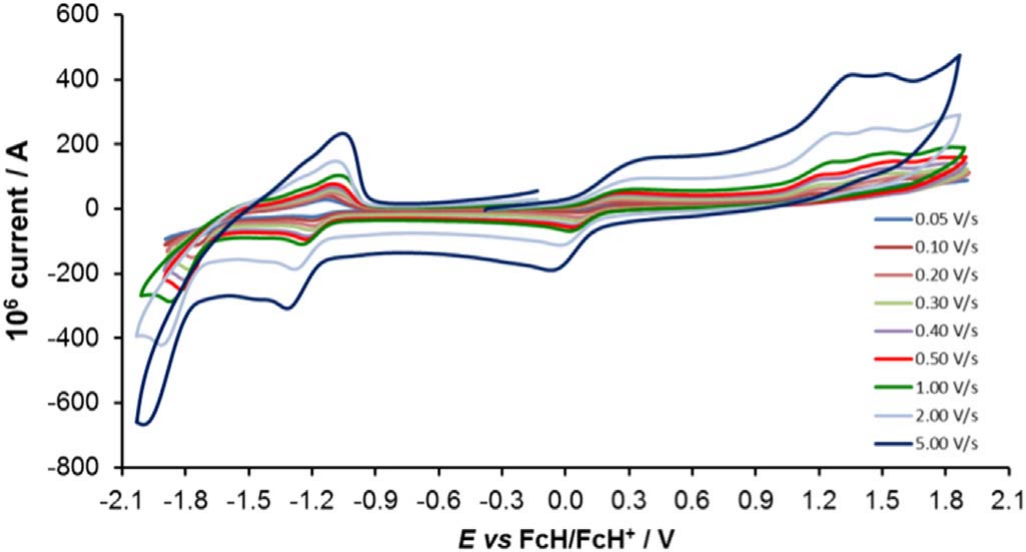
Cyclic voltammograms of complex 2 at scan rates of 0.05 V s^−1^ (lowest peak current) −5.00 V s^−1^ (highest peak current). All scans initiated in the positive direction.

**Fig. 4:**
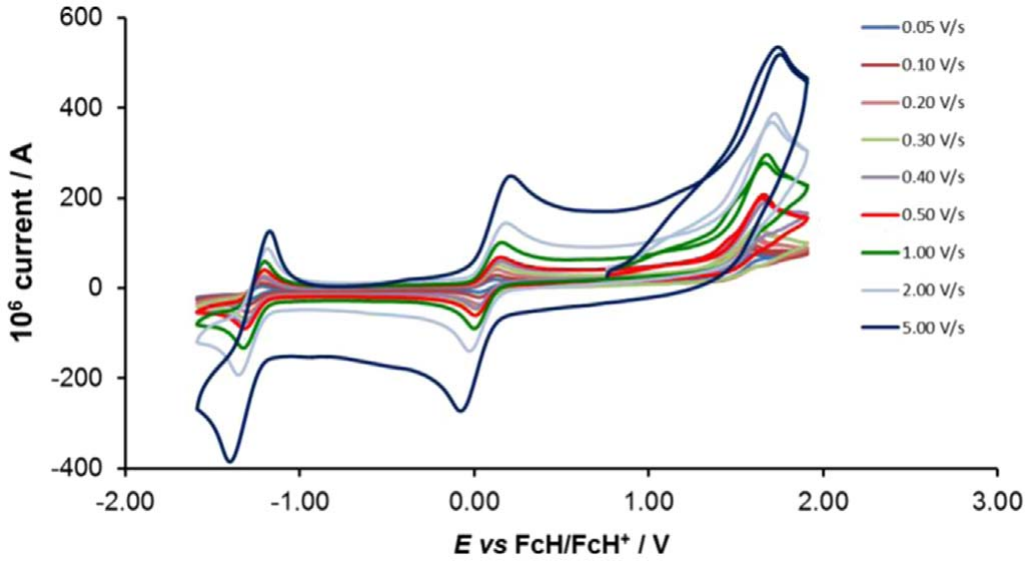
Cyclic voltammograms of complex 3 at scan rates of 0.05 V s^−1^ (lowest peak current) −5.00 V s^−1^ (highest peak current). All scans initiated in the positive direction.

**Fig. 5:**
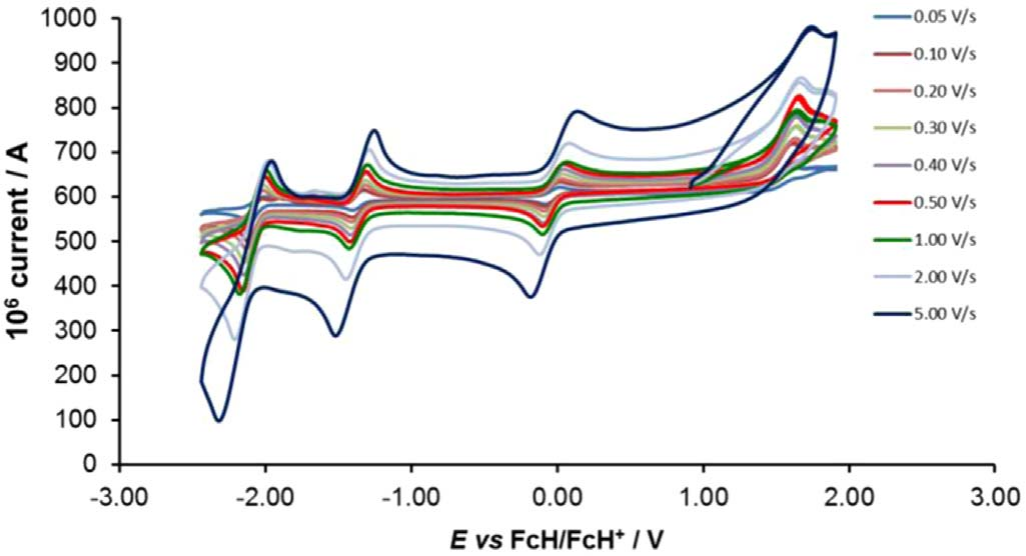
Cyclic voltammograms of complex 4 at scan rates of 0.05 V s^−1^ (lowest peak current) −5.00 V s^−1^ (highest peak current). All scans initiated in the positive direction.

**Fig. 6:**
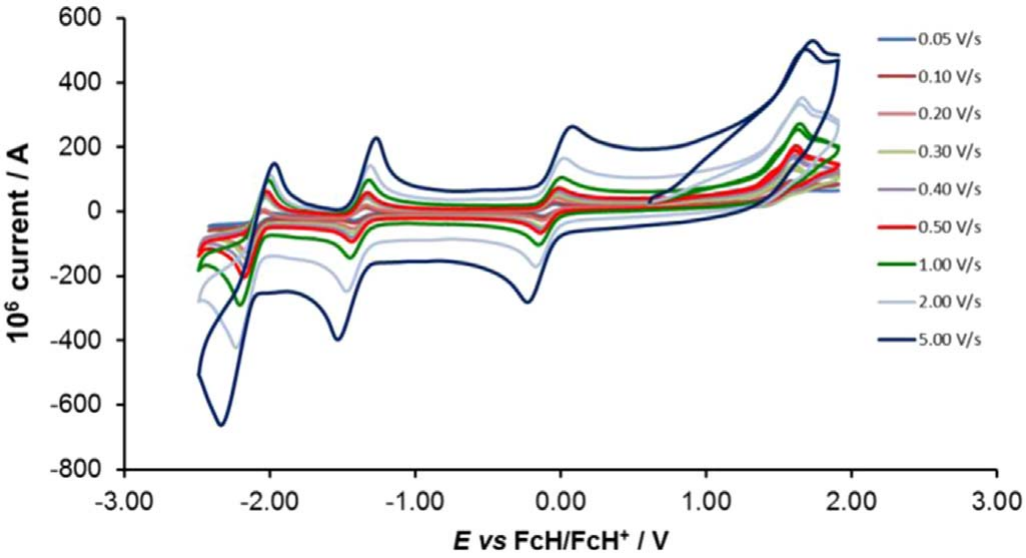
Cyclic voltammograms of complex 5 at scan rates of 0.05 V s^−1^ (lowest peak current) −5.00 V s^−1^ (highest peak current). All scans initiated in the positive direction.

**Fig. 7:**
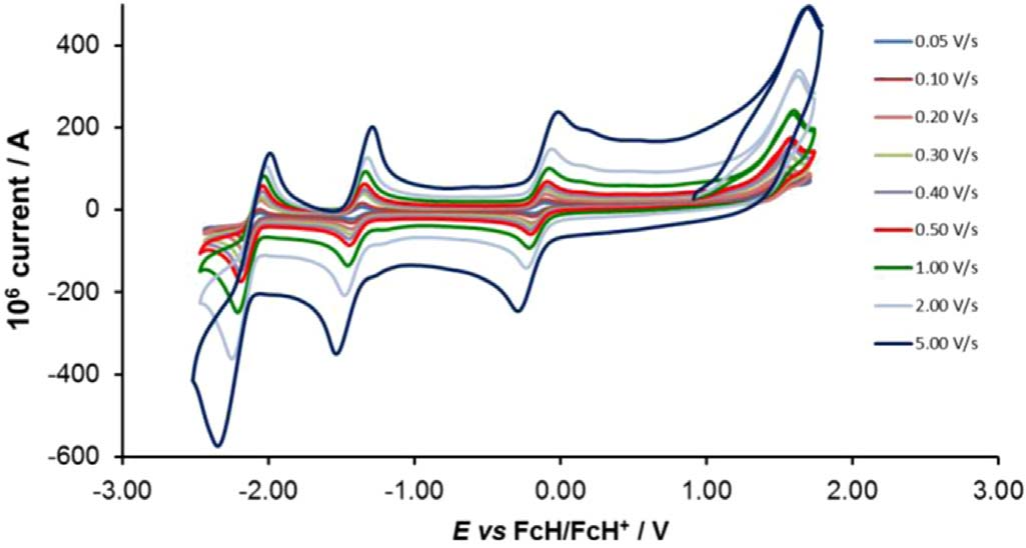
Cyclic voltammograms of complex 6 at scan rates of 0.05 V s^−1^ (lowest peak current) −5.00 V s^−1^ (highest peak current). All scans initiated in the positive direction.

**Fig. 8:**
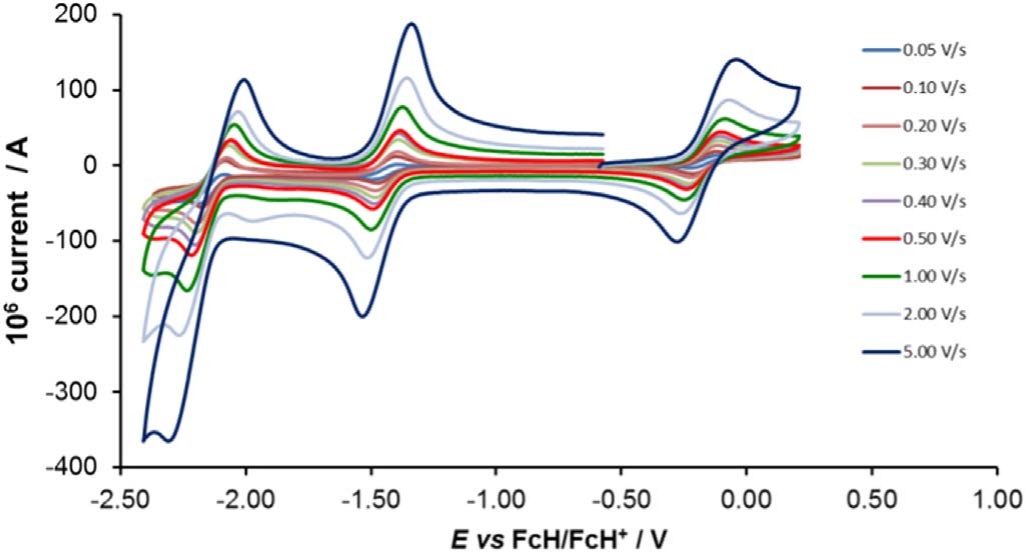
Cyclic voltammograms of complex 7 at scan rates of 0.05 V s^−1^ (lowest peak current) −5.00 V s^−1^ (highest peak current). All scans initiated in the positive direction.

**Fig. 9:**
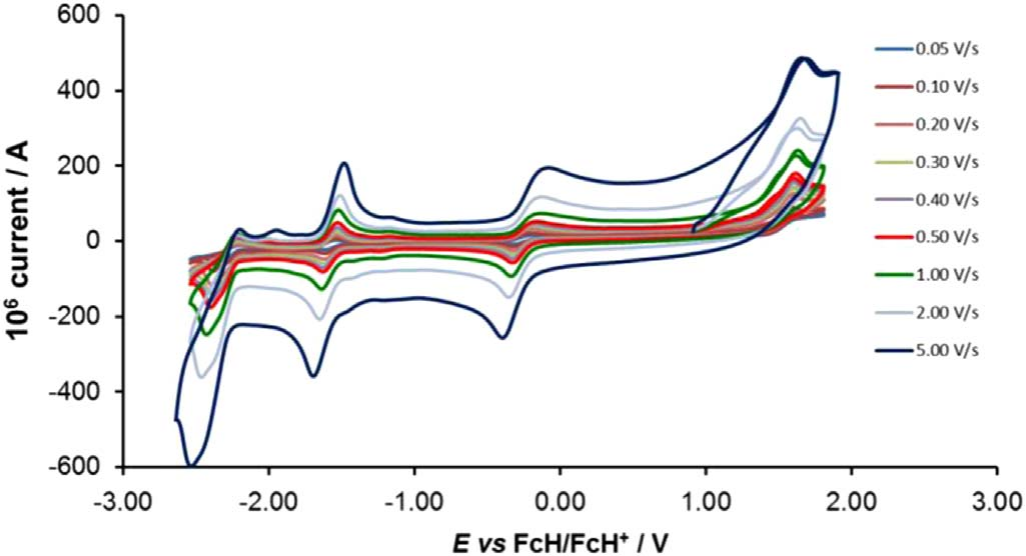
Cyclic voltammograms of complex 8 at scan rates of 0.05 V s^−1^ (lowest peak current) −5.00 V s^−1^ (highest peak current). All scans initiated in the positive direction.

Cyclic voltammograms of the complexes **1**–**8**, are presented in Fig. 2, Fig. 3, Fig. 4, Fig. 5, Fig. 6, Fig. 7, Fig. 8, Fig. 9 and tabulated in Table 1, Table 2, Table 3, Table 4, Table 5, Table 6, Table 7, Table 8. The electrochemical data is obtained in CH_3_CN for *ca* 0.002 mol dm^−3^ (or saturated) analyte solution. Complexes **3**–**8**, all have three reversible peaks, namely the Co^III/II^ redox couple (peak 1), the Co^II/I^ redox couple (peak 2) and the ligand reduction peak (peak 3). For complex **2** the ligand reduction peak (peak 3) is irreversible and for complex **1** the irreversible peak 2 is NO_2_-ligand based. Data at scan rates 0.05–5.00 V s^−1^ are provided. Data for the irreversible anionic nitrate oxidation peak at *ca* 1.63 V *vs* FcH/FcH^+^, is not included in the tables. The data obtained in this study, compare good with available published data on some of the complexes, namely complex **2** [5], complex **4** [6], [7], [8], [9], complex **7** [9] and complex **8** [9], obtained under different experimental conditions (different solvents, scan rates and supporting electrolytes).

**Table 1:** Electrochemical data (potential in V *vs* FcH/FcH^+^) in CH_3_CN for *ca* 0.002 mol dm^−3^ of complex 1 at indicated scan rates in V s^−1^. Peak 1 is the Co^III/II^ redox couple.

	Scan rate/Vs^−1^	*E*_pa_/V *vs* FcH/FcH^+^	*E*_pc_/V *vs* FcH/FcH^+^	*E*°׳/V *vs* FcH/FcH^+^	Δ*E*/V
Peak 1	0.05	0.275	0.110	0.165	0.193
	0.10	0.290	0.115	0.175	0.203
	0.20	0.320	0.120	0.200	0.220
	0.30	0.290	0.110	0.180	0.200
	0.40	0.300	0.110	0.190	0.205
	0.50	0.300	0.105	0.195	0.203
	1.00	0.330	0.095	0.235	0.213
	2.00	0.415	0.050	0.365	0.233
	5.00	0.560	0.005	0.555	0.283

**Table 2:** Electrochemical data (potential in V *vs* FcH/FcH^+^) in CH_3_CN for *ca* 0.002 mol dm^−3^ of complex 2 at indicated scan rates in V s^−1^. Peak 1 is the Co^III/II^ redox couple, peak 2 the Co^II/I^ redox couple.

	Scan rate/V s^−1^	*E*_pa_/V *vs* FcH/FcH^+^	*E*_pc_/V *vs* FcH/FcH^+^	*E*°′/V *vs* FcH/FcH^+^	Δ*E*/V
Peak 1	0.10	0.200	0.070	0.130	0.135
	0.20	0.260	0.070	0.190	0.165
	0.30	0.250	0.050	0.200	0.150
	0.40	0.270	0.050	0.220	0.160
	0.50	0.260	0.030	0.230	0.145
	1.00	0.300	0.020	0.280	0.160
	2.00	0.380	0.000	0.380	0.190
	5.00	0.400	−0.070	0.470	0.165
Peak 2	0.10	−1.120	−1.200	0.080	−1.160
	0.20	−1.105	−1.195	0.090	−1.150
	0.30	−1.105	−1.200	0.095	−1.153
	0.40	−1.100	−1.210	0.110	−1.155
	0.50	−1.100	−1.225	0.125	−1.163
	1.00	−1.080	−1.250	0.170	−1.165
	2.00	−1.075	−1.260	0.185	−1.168
	5.00	−1.060	−1.310	0.250	−1.185

**Table 3:** Electrochemical data (potential in V *vs* FcH/FcH^+^ and current in A) in CH_3_CN for *ca* 0.002 mol dm^−3^ of complex 3 at indicated scan rates in V s^−1^. Peak 1 is the Co^III/II^ redox couple, peak 2 the Co^II/I^ redox couple.

	Scan rate/V s^−1^	*E*_pa_/V *vs* FcH/FcH^+^	*E*_pc_/V *vs* FcH/FcH^+^	*E*°׳/V *vs* FcH/FcH^+^	Δ*E*/V	10^6^*I*_pa_/A	*I*_pc_/*I*_pa_
Peak 1	0.05	0.108	0.034	0.071	0.074	17.5	1.0
	0.10	0.122	0.032	0.077	0.090	26.0	1.0
	0.20	0.122	0.034	0.078	0.088	39.5	1.0
	0.30	0.136	0.022	0.079	0.114	44.2	1.0
	0.40	0.142	0.016	0.079	0.126	51.4	1.1
	0.50	0.144	0.010	0.077	0.134	63.0	1.1
	1.00	0.152	0.004	0.078	0.148	87.5	1.1
	2.00	0.176	−0.024	0.076	0.200	117.0	1.1
	5.00	0.202	−0.070	0.066	0.272	200.0	1.0
Peak 2	0.05	−1.220	−1.292	−1.256	0.072	17.0	0.9
	0.10	−1.216	−1.296	−1.256	0.080	25.0	1.0
	0.20	−1.218	−1.292	−1.255	0.074	37.0	1.0
	0.30	−1.210	−1.300	−1.255	0.090	42.3	1.1
	0.40	−1.210	−1.314	−1.262	0.104	53.0	1.0
	0.50	−1.208	−1.314	−1.261	0.106	64.8	1.0
	1.00	−1.206	−1.318	−1.262	0.112	98.0	1.0
	2.00	−1.192	−1.346	−1.269	0.154	135.0	1.0
	5.00	−1.180	−1.398	−1.289	0.218	192.0	1.2

**Table 4:** Electrochemical data (potential in V *vs* FcH/FcH^+^ and current in A) in CH_3_CN for *ca* 0.002 mol dm^−3^ of complex 4 at indicated scan rates in V s^−1^. Peak 1 is the Co^III/II^ redox couple, peak 2 the Co^II/I^ redox couple and peak 3 the ligand reduction peak.

	Scan rate/Vs^−1^	*E*_pa_/V *vs* FcH/FcH^+^	*E*_pc_/V *vs* FcH/FcH^+^	*E*°׳/V *vs* FcH/FcH^+^	Δ*E*/V	10^6^*I*_pa_/A	*I*_pc_/*I*_pa_
Peak 1	0.05	0.012	−0.076	−0.032	0.088	17.8	1.12
	0.10	0.010	−0.082	−0.036	0.092	29.5	1.08
	0.20	0.026	−0.082	−0.028	0.108	34.5	1.10
	0.30	0.032	−0.086	−0.027	0.118	44.0	1.07
	0.40	0.038	−0.092	−0.027	0.130	51.0	1.14
	0.50	0.040	−0.094	−0.027	0.134	62.0	1.13
	1.00	0.060	−0.096	−0.018	0.156	59.0	1.20
	2.00	0.074	−0.116	−0.021	0.190	82.0	1.20
	5.00	0.130	−0.180	−0.025	0.310	136.0	1.07
Peak 2	0.05	−1.326	−1.398	−1.362	0.072	22.8	0.99
	0.10	−1.328	−1.404	−1.366	0.076	32.3	1.04
	0.20	−1.322	−1.404	−1.363	0.082	41.3	0.99
	0.30	−1.318	−1.410	−1.364	0.092	54.0	0.98
	0.40	−1.316	−1.414	−1.365	0.098	60.0	1.08
	0.50	−1.314	−1.416	−1.365	0.102	76.0	1.01
	1.00	−1.304	−1.424	−1.364	0.120	81.0	0.98
	2.00	−1.292	−1.446	−1.369	0.154	99.0	1.19
	5.00	−1.256	−1.516	−1.386	0.260	150.0	1.20
Peak 3	0.05	−2.026	−2.104	−2.065	0.078	21.0	1.93
	0.10	−2.026	−2.114	−2.070	0.088	45.0	1.51
	0.20	−2.018	−2.130	−2.074	0.112	56.0	1.47
	0.30	−2.016	−2.140	−2.078	0.124	75.0	1.41
	0.40	−2.010	−2.144	−2.077	0.134	91.0	1.43
	0.50	−2.008	−2.152	−2.080	0.144	114.0	1.37
	1.00	−1.998	−2.172	−2.085	0.174	108.0	1.41
	2.00	−1.984	−2.206	−2.095	0.222	148.0	1.44
	5.00	−1.964	−2.316	−2.140	0.352	200.7	1.5

**Table 5:** Electrochemical data (potential in V *vs* FcH/FcH^+^ and current in A) in CH_3_CN for *ca* 0.002 mol dm^−3^ of complex 5 at indicated scan rates in V s^−1^. Peak 1 is the Co^III/II^ redox couple, peak 2 the Co^II/I^ redox couple and peak 3 the ligand reduction peak.

	Scan rate/V s^−1^	*E*_pa_/V *vs* FcH/FcH^+^	*E*_pc_/V *vs* FcH/FcH^+^	*E*°׳/V *vs* FcH/FcH^+^	Δ*E*/V	10^6^*I*_pa_/A	*I*_pc_/*I*_pa_
Peak 1	0.05	−0.046	−0.118	−0.082	0.072	22.0	1.0
	0.10	−0.042	−0.116	−0.079	0.074	25.0	1.1
	0.20	−0.036	−0.122	−0.079	0.086	37.5	1.0
	0.30	−0.028	−0.126	−0.077	0.098	45.0	1.1
	0.40	−0.026	−0.130	−0.078	0.104	54.5	1.1
	0.50	−0.018	−0.134	−0.076	0.116	62.0	1.3
	1.00	0.002	−0.144	−0.071	0.146	84.0	1.1
	2.00	0.014	−0.166	−0.076	0.180	120.0	1.1
	5.00	0.070	−0.222	−0.076	0.292	188.0	1.1
Peak 2	0.05	−1.350	−1.418	−1.384	0.068	22.5	0.9
	0.10	−1.348	−1.414	−1.381	0.066	25.0	1.0
	0.20	−1.342	−1.422	−1.382	0.080	39.5	1.0
	0.30	−1.338	−1.428	−1.383	0.090	48.0	1.0
	0.40	−1.332	−1.430	−1.381	0.098	56.0	1.1
	0.50	−1.332	−1.436	−1.384	0.104	69.0	1.0
	1.00	−1.322	−1.448	−1.385	0.126	104.0	1.0
	2.00	−1.312	−1.470	−1.391	0.158	144.0	1.0
	5.00	−1.274	−1.528	−1.401	0.254	230.0	1.0
Peak 3	0.05	−2.046	−2.128	−2.087	0.082	37.1	1.3
	0.10	−2.050	−2.138	−2.094	0.088	45.8	1.3
	0.20	−2.046	−2.156	−2.101	0.110	61.0	1.4
	0.30	−2.034	−2.160	−2.097	0.126	84.0	1.3
	0.40	−2.030	−2.168	−2.099	0.138	105.0	1.3
	0.50	−2.026	−2.174	−2.100	0.148	124.0	1.2
	1.00	−2.012	−2.204	−2.108	0.192	172.0	1.3
	2.00	−2.002	−2.228	−2.115	0.226	214.0	1.3
	5.00	−1.974	−2.332	−2.153	0.358	250.0	1.6

**Table 6:** Electrochemical data (potential in V *vs* FcH/FcH^+^ and current in A) in CH_3_CN for *ca* 0.002 mol dm^−3^ of complex 6 at indicated scan rates in V s^−1^. Peak 1 is the Co^III/II^ redox couple, peak 2 the Co^II/I^ redox couple and peak 3 the ligand reduction peak.

	Scan rate/V s^−1^	*E*_pa_/V *vs* FcH/FcH^+^	*E*_pc_/V *vs* FcH/FcH^+^	*E*°׳/V *vs* FcH/FcH^+^	Δ*E*/V	10^6^*I*_pa_/A	*I*_pc_/*I*_pa_
Peak 1	0.05	−0.118	−0.180	−0.149	0.062	11.0	0.9
	0.10	−0.114	−0.188	−0.151	0.074	23.0	1.0
	0.20	−0.108	−0.188	−0.148	0.080	33.0	1.0
	0.30	−0.104	−0.192	−0.148	0.088	42.0	1.0
	0.40	−0.096	−0.198	−0.147	0.102	49.0	1.0
	0.50	−0.094	−0.198	−0.146	0.104	57.0	1.0
	1.00	−0.086	−0.210	−0.148	0.124	80.0	1.0
	2.00	−0.068	−0.228	−0.148	0.160	112.0	1.0
	5.00	−0.026	−0.288	−0.157	0.262	173.0	1.0
Peak 2	0.05	−1.366	−1.428	−1.397	0.062	13.4	0.9
	0.10	−1.360	−1.430	−1.395	0.070	25.7	0.9
	0.20	−1.352	−1.432	−1.392	0.080	43.5	0.8
	0.30	−1.352	−1.436	−1.394	0.084	48.0	0.9
	0.40	−1.344	−1.444	−1.394	0.100	56.0	0.9
	0.50	−1.346	−1.444	−1.395	0.098	63.0	1.0
	1.00	−1.340	−1.454	−1.397	0.114	93.0	1.0
	2.00	−1.326	−1.476	−1.401	0.150	127.0	0.9
	5.00	−1.292	−1.532	−1.412	0.240	204.0	1.0
Peak 3	0.05	−2.064	−2.146	−2.105	0.082	29.5	1.3
	0.10	−2.068	−2.152	−2.110	0.084	41.2	1.3
	0.20	−2.058	−2.162	−2.110	0.104	60.5	1.3
	0.30	−2.054	−2.172	−2.113	0.118	72.5	1.3
	0.40	−2.046	−2.190	−2.118	0.144	87.0	1.3
	0.50	−2.046	−2.184	−2.115	0.138	103.0	1.3
	1.00	−2.034	−2.206	−2.120	0.172	144.0	1.3
	2.00	−2.022	−2.246	−2.134	0.224	188.0	1.3
	5.00	−1.994	−2.342	−2.168	0.348	238.0	1.5

**Table 7:** Electrochemical data (potential in V *vs* FcH/FcH^+^ and current in A) in CH_3_CN for *ca* 0.002 mol dm^−3^ of complex 7 at indicated scan rates in V s^−1^. Peak 1 is the Co^III/II^ redox couple, peak 2 the Co^II/I^ redox couple and peak 3 the ligand reduction peak.

	Scan rate/V s^−1^	*E*_pa_/V *vs* FcH/FcH^+^	*E*_pc_/V *vs* FcH/FcH^+^	*E*°׳/V *vs* FcH/FcH^+^	Δ*E*/V	10^6^*I*_pa_/A	*I*_pc_/*I*_pa_
Peak 1	0.05	−0.128	−0.216	−0.172	0.088	12.5	1.0
	0.10	−0.127	−0.213	−0.170	0.086	16.2	1.0
	0.20	−0.121	−0.218	−0.170	0.097	23.3	1.0
	0.30	−0.116	−0.227	−0.172	0.111	29.0	1.0
	0.40	−0.116	−0.230	−0.173	0.114	35.0	0.9
	0.50	−0.109	−0.234	−0.172	0.125	41.0	0.9
	1.00	−0.091	−0.244	−0.168	0.153	55.5	0.9
	2.00	−0.080	−0.256	−0.168	0.176	78.0	0.8
	5.00	−0.053	−0.272	−0.163	0.219	130.5	0.8
Peak 2	0.05	−1.401	−1.486	−1.444	0.085	15.4	1.0
	0.10	−1.402	−1.473	−1.438	0.071	20.5	0.9
	0.20	−1.398	−1.480	−1.439	0.082	29.0	0.9
	0.30	−1.393	−1.482	−1.438	0.089	32.5	1.1
	0.40	−1.391	−1.485	−1.438	0.094	42.8	1.0
	0.50	−1.388	−1.491	−1.440	0.103	51.0	0.9
	1.00	−1.376	−1.497	−1.437	0.121	75.0	0.9
	2.00	−1.360	−1.515	−1.438	0.155	109.0	0.9
	5.00	−1.340	−1.533	−1.437	0.193	179.0	0.9
Peak 3	0.05	−2.088	−2.186	−2.137	0.098	28.2	1.1
	0.10	−2.086	−2.174	−2.130	0.088	29.4	1.4
	0.20	−2.079	−2.194	−2.137	0.115	38.2	1.5
	0.30	−2.071	−2.195	−2.133	0.124	47.8	1.4
	0.40	−2.066	−2.199	−2.133	0.133	52.0	1.6
	0.50	−2.063	−2.215	−2.139	0.152	54.4	1.7
	1.00	−2.049	−2.232	−2.141	0.183	69.4	1.8
	2.00	−2.037	−2.263	−2.150	0.226	85.8	1.9
	5.00	−2.010	−2.303	−2.157	0.293	133.4	2.0

**Table 8:** Electrochemical data (potential in V *vs* FcH/FcH^+^ and current in A) in CH_3_CN for *ca* 0.002 mol dm^−3^ of complex 8 at indicated scan rates in V s^−1^. Peak 1 is the Co^III/II^ redox couple, peak 2 the Co^II/I^ redox couple and peak 3 the ligand reduction peak.

	Scan rate/Vs^−1^	*E*_pa_/V *vs* FcH/FcH^+^	*E*_pc_/V *vs* FcH/FcH^+^	*E*°׳/V *vs* FcH/FcH^+^	Δ*E*/V	10^6^*I*_pa_/A	*I*_pc_/*I*_pa_
Peak 1	0.05	−0.216	−0.314	−0.265	0.098	13.2	1.0
	0.10	−0.210	−0.320	−0.263	0.110	19.5	1.1
	0.20	−0.210	−0.314	−0.262	0.104	28.5	1.1
	0.30	−0.200	−0.316	−0.258	0.116	34.5	1.2
	0.40	−0.192	−0.318	−0.255	0.126	39.0	1.3
	0.50	−0.182	−0.324	−0.253	0.142	43.0	1.4
	1.00	−0.166	−0.326	−0.246	0.160	56.0	1.4
	2.00	−0.142	−0.336	−0.239	0.194	86.0	1.3
	5.00	−0.136	−0.348	−0.242	0.212	137.0	1.3
Peak 2	0.05	−1.546	−1.610	−1.578	0.064	14.0	1.0
	0.10	−1.560	−1.615	−1.581	0.055	20.8	1.0
	0.20	−1.542	−1.616	−1.579	0.074	31.5	1.0
	0.30	−1.540	−1.616	−1.578	0.076	39.5	1.0
	0.40	−1.534	−1.622	−1.578	0.088	45.0	1.0
	0.50	−1.532	−1.628	−1.580	0.096	53.5	1.1
	1.00	−1.530	−1.626	−1.578	0.096	81.0	1.0
	2.00	−1.526	−1.634	−1.580	0.108	117.5	1.0
	5.00	−1.514	−1.646	−1.580	0.132	190.0	1.1
Peak 3	0.05	−2.196	−2.382	−2.289	0.186	10.0	4.0
	0.10	−2.202	−2.298	−2.250	0.096	8.0	6.1
	0.20	−2.204	−2.392	−2.298	0.188	12.0	6.3
	0.30	−2.218	−2.396	−2.307	0.178	19.0	4.4
	0.40	−2.218	−2.402	−2.310	0.184	22.0	4.6
	0.50	−2.222	−2.400	−2.311	0.178	20.0	5.6
	1.00	−2.222	−2.428	−2.325	0.206	22.0	7.1
	2.00	−2.218	−2.462	−2.340	0.244	22.0	10.3
	5.00	−2.204	−2.532	−2.368	0.328	27.0	13.0

For Co(5-NO_2_-phen)_3_^2+^ complex 1, see Fig. 2 and Table 1.

For Co(4,7-di-Cl-phen)_3_^2+^ complex 2, see Fig. 3 and Table 2.

For Co(5-Cl-phen)_3_^2+^ complex 3, see Fig. 4 and Table 3.

For Co(phen)_3_^2+^ complex 4, see Fig. 5 and Table 4.

For Co(5-Me-phen)_3_^2+^ complex 5, see Fig. 6 and Table 5.

For Co(5,6-Me-phen)_3_^2+^ complex 6, see Fig. 7 and Table 6.

For Co(5-NH_2_-phen)_3_^2+^ complex 7, see Fig. 8 and Table 7.

For Co(3,4,7,8-Me-phen)_3_^2+^ complex 8, see Fig. 9 and Table 8.

## 2 Experimental design, materials, and methods

Electrochemical studies by means of cyclic voltammetry (CV) were performed either on 0.002 mol dm^−3^ or on saturated compound solutions of the complexes in dry acetonitrile, containing 0.1 mol dm^−3^ tetra-*n*-butylammoniumhexafluorophosphate ([^n^(Bu_4_)N][PF_6_]) as supporting electrolyte, under a blanket of purified argon, at 25 °C, utilizing a BAS 100B/W electrochemical analyzer. A three-electrode cell was used, with a glassy carbon (surface area 7.07 × 10^−6^ m^2^) working electrode, Pt auxiliary electrode and a Ag/Ag^+^ (0.010 mol dm^−3^ AgNO_3_ in CH_3_CN) reference electrode [10], mounted on a Luggin capillary [11]. Scan rates for the CVs were 0.050─5.000 V s^−1^. Successive experiments under the same experimental conditions showed that all oxidation and reduction potentials were reproducible within 0.010 V under our experimental conditions. Electrochemical data in Table 1, Table 2, Table 3, Table 4, Table 5, Table 6, Table 7, Table 8 is obtained from the cyclic voltammograms presented in Fig. 2, Fig. 3, Fig. 4, Fig. 5, Fig. 6, Fig. 7, Fig. 8, Fig. 9. Potentials tabulated are referenced against the FcH/FcH^+^ couple, as suggested by IUPAC [12].
