# Supplementary material for: Electrochemical data of Co(II) complexes containing phenanthroline functionalized ligands
Source: Data Brief. 2018 Oct 19;21:866–77. doi: 10.1016/j.dib.2018.10.046 (PMC6223223; doi:10.1016/j.dib.2018.10.046)
Supplement: Supplementary file 1 — Transparency document [file mmc1.docx]

The authors declare that there is no conflict of interest regarding the publication of this article
